# Evaluating Carotenoids Intake of Pregnant Women: A FFQ-Based Approach to Dietary Patterns

**DOI:** 10.3390/nu18121999

**Published:** 2026-06-19

**Authors:** Andreea-Maria Mitran, Alina-Delia Popa, Catalin-Mihail Chiru, Cornelia Mircea, Ionut Iulian Lungu, Ioana-Cezara Caba, Andreea Lungu, Cristina Arsene, Dumitru Gafitanu, Florina Crivoi, Monica Hancianu, Cristina Elena Dobre, Oana Cioanca

**Affiliations:** 1 Faculty of Pharmacy, Grigore T. Popa University of Medicine and Pharmacy Iasi, 16 Universitatii Street, 700115 Iasi, Romania; andreea-maria.mitran@umfiasi.ro (A.-M.M.); cornelia.mircea@umfiasi.ro (C.M.); ioana-cezara.caba@umfiasi.ro (I.-C.C.); florina.crivoi@umfiasi.ro (F.C.); monica.hancianu@umfiasi.ro (M.H.); oana.cioanca@umfiasi.ro (O.C.); 2 Faculty of Medicine, Grigore T. Popa University of Medicine and Pharmacy Iasi, 16 Universitatii Street, 700115 Iasi, Romania; alina.popa@umfiasi.ro (A.-D.P.); andreea-lungu@umfiasi.ro (A.L.); cristina.arsene@umfiasi.ro (C.A.); dumitru.gafitanu@umfiasi.ro (D.G.); 3 Department of Computer Science and Information Technology, National University of Science and Technology Politehnica Bucharest, Splaiul Independentei No. 313, 011061 Bucharest, Romania; catalin.chiru@upb.ro; 4 Institute of Psychiatry “Socola” Iasi, Str. Bucium 36, 700282 Iasi, Romania; cedobre@yahoo.com

**Keywords:** carotenoids, pregnancy, maternal diet, dietary assessment, dietary patterns, diet quality

## Abstract

Background: Pregnancy is a vital period during which maternal nutrition profoundly influences both maternal health and fetal development. Carotenoids, predominantly found in fruits and vegetables, are bioactive compounds that enhance antioxidant defenses and facilitate vitamin A metabolism throughout pregnancy. However, assessing carotenoids intake presents challenges due to the lack of dietary assessment tools capable of quantifying individual carotenoids, coupled with limited data from populations in Eastern Europe. Methods: A cross-sectional study involving 621 pregnant women in Romania was conducted to estimate dietary carotenoids intake and investigate associations with dietary patterns and overall diet quality. Dietary data were obtained using the EPIC Food Frequency Questionnaire (EPIC-FFQ), adapted for Romanian populations. A dedicated carotenoid estimation model was developed utilizing the USDA Carotenoid Database. Principal component analysis (PCA) was employed to identify dietary patterns, and diet quality was evaluated using the Diet Quality Index during Pregnancy (DQI-P). Results: The findings revealed significant individual variability. The median intake was highest for β-carotene (2464 μg), and lycopene (1664 μg), followed by lutein and zeaxanthin (908 μg), α-carotene (615 μg), and β-cryptoxanthin (121 μg). The Vegetable-meal pattern exhibited the strongest positive correlation with carotenoids intake, whereas the Energy-dense pattern was primarily associated with vitamin E and tocopherols/tocotrienols, and the Mixed pattern with vitamins A and D. Higher DQI-P scores were consistently correlated with increased carotenoids consumption. Conclusions: Overall, maternal carotenoids intake during pregnancy was frequently insufficient and showed considerable variation among women. A diet rich in vegetables and higher overall diet quality were associated with elevated carotenoids intake levels. These findings enhance the understanding of dietary carotenoids intake among pregnant women in Eastern Europe.

## 1. Introduction

Maternal nutrition is one of the major factors influencing pregnancy outcomes and fetal development. Adequate nutrition is critically important during pregnancy because it supports maternal health, affects pregnancy progression and outcomes, influences fetal development, and has lasting effects on the child’s health, both postnatally and throughout life [1,2]. In addition to essential dietary components required for maternal and fetal physiology, increasing attention has been directed toward plant-derived bioactive compounds that may contribute to antioxidant defenses, immune regulation, and fetal development.

Carotenoids are fat-soluble pigments synthesized by plants and certain microorganisms [2]. More than 700 varieties have been recognized and are generally classified into two categories: carotenes, such as β-carotene, α-carotene, and lycopene; and oxygenated derivatives known as xanthophylls, including lutein, zeaxanthin, β-cryptoxanthin, and astaxanthin [2]. Although only approximately 50 carotenoids are typically present in the human diet, approximately 95% of those found in human plasma comprise six primary compounds: α-carotene, β-carotene, β-cryptoxanthin, lycopene, lutein, and zeaxanthin [2].

Carotenoids have been associated with antioxidant and anti-inflammatory effects [3,4]. They are generally classified into provitamin A and non-provitamin A carotenoids. β-carotene, α-carotene, and β-cryptoxanthin can be converted to retinol by enzymatic cleavage, primarily mediated by β-carotene monooxygenase 1 (BCMO1), thereby contributing to vitamin A levels [5]. However, the efficiency of this conversion is influenced by several factors, including diet composition, nutritional status, food matrix, and genetic variability, and may be relatively limited in humans [6,7].

Vitamin A plays an essential role in embryonic development, immune function, epithelial differentiation, and visual processes [5,6]. During pregnancy, maintaining an adequate vitamin A status is particularly important due to increased maternal and fetal demands. However, excessive intake of preformed vitamin A has been associated with teratogenic effects and congenital malformations, especially during early embryogenesis [7]. Consequently, provitamin A carotenoids obtained from fruits and vegetables are generally considered a safer dietary source because their conversion into retinol is physiologically regulated and less likely to result in toxicity [8]. Nevertheless, excessive vitamin A intake during pregnancy can also have adverse effects. Intakes of over 10,000 IU of preformed vitamin A per day pose teratogenic risks, with malformations observed in children whose mothers consume over 25,000 IU of preformed vitamin A during pregnancy [9].

Pregnancy is a particularly significant period for assessing dietary carotenoids intake, as maternal nutrition substantially influences maternal health, pregnancy progression, fetal development, and long-term offspring outcomes [1,2]. During this phase, a woman’s physiology undergoes notable metabolic changes alongside increased oxidative stress, partially attributable to heightened placental mitochondrial activity and reactive oxygen species, thereby potentially increasing the importance of dietary antioxidants [2,10]. Moreover, maintaining adequate vitamin A levels is essential for embryonic and fetal growth due to their roles in cellular differentiation, epithelial development, immune response, and developmental signaling pathways [5,6]. Given that many carotenoids function as provitamin A and may enhance antioxidant defenses, evaluating their intake during pregnancy can provide valuable insights into maternal nutritional patterns during this physiologically demanding period.

Also, pregnancy is characterized by increased oxidative stress, with enhanced placental mitochondrial activity and increased production of reactive oxygen species (ROS) [10]. However, if oxidative stress exceeds the placenta’s antioxidant mechanisms, the resulting oxidative damage may contribute to numerous pregnancy complications such as pregnancy-induced hypertension, preeclampsia, and other placental diseases [11,12]. Dietary carotenoids have important antioxidant activity due to their conjugated double bonds [13]. β-carotene, α-carotene, and zeaxanthin, in particular, are among the most effective quenchers of singlet molecular oxygen, ROS, and peroxyl radicals. Moreover, these molecules effectively deactivate singlet molecular oxygen and radical-generating species via electronic excitation [14]. Therefore, dietary carotenoids may contribute to antioxidant defense mechanisms involved in healthy pregnancy progression.

Although carotenoids are associated with potential health benefits, current evidence regarding their effects during pregnancy remains inconclusive and varies across studies [3,10]. The biological activity of carotenoids is influenced by factors such as dosage, source, smoking status, and overall dietary habits. While the consumption of carotenoids through fruits and vegetables is generally considered safe, high-dose β-carotene supplementation has been linked to adverse effects in certain populations, notably smokers, among whom an increased risk of lung cancer has been observed [11]. Furthermore, precise measurement of carotenoids intake presents challenges due to regional disparities in food composition data, agricultural practices, food processing methods, and dietary assessment techniques [12,13].

While carotenoids are recognized for their health benefits and have been studied as dietary components, intake among pregnant women remains generally under-researched. A major limitation in carotenoids intake research is the lack of accessible dietary assessment tools that also measure individual carotenoid consumption. Most food frequency questionnaires (FFQs) assess intake of preformed vitamin A and total carotenoids but do not differentiate among individual carotenoids, such as lycopene, zeaxanthin, or β-cryptoxanthin. Conversely, although targeted FFQs exist that measure specific carotenoids intake, they often do so at the expense of other minerals and vitamin profiles. Researchers often must choose between evaluating carotenoid variability and performing comprehensive dietary assessments.

Therefore, this paper aims to: (i) assess carotenoids intake using an existing, validated food frequency questionnaire, (ii) evaluate the carotenoids intake of pregnant women, and (iii) examine the relationship between carotenoids intake and maternal dietary patterns.

## 2. Materials and Methods

### 2.1. Study Design

A cross-sectional study was conducted in 2023 using a convenience sample of pregnant women admitted for delivery at the Elena Doamna Regional Obstetrics and Gynecology Hospital in Iasi, Romania. Exclusion criteria were: declining to participate, obstetric or psychiatric diagnoses, or any disease that might adversely affect their understanding of the study objectives or their ability to provide accurate information.

The required sample size was calculated using G*Power 3.1 [15]. Assuming a two-tailed correlation of r = 0.15, α = 0.05, and a statistical power of 95%, the minimum required sample size for correlation analyses was 571 participants. Additionally, a one-way ANOVA model including four groups and assuming a small-to-moderate effect size (f = 0.20; η^2^ ≈ 0.039) indicated a minimum required sample size of 436 participants.

A total of 700 pregnant women were invited to participate in the study. Questionnaires with substantial missing data, participant withdrawal, or implausible estimated daily energy intake values derived from the food frequency questionnaire (<800 kcal/day or >3500 kcal/day) were excluded from the final analysis. After data cleaning, the final analytical sample comprised 621 participants, exceeding the estimated minimum sample size and providing sufficient statistical power for both correlation and group-comparison analyses.

### 2.2. Data Collection

Socioeconomic data. Data regarding maternal age, area of residence (urban/rural), monthly household income, relationship status, parity, and educational level were collected using a structured questionnaire. Household income was categorized as low (<600 EUR/month), lower-middle (600–1000 EUR/month), upper-middle (1000–1600 EUR/month), and high (>1600 EUR/month).

Dietary Assessment. Dietary intake was assessed using the EPIC-Norfolk Food Frequency Questionnaire (EPIC-FFQ), previously translated and adapted for Romanian populations [16,17]. The semiquantitative FFQ assesses habitual dietary intake using 130 food and beverage items. Participants were asked to report their usual dietary intake during pregnancy, reflecting habitual rather than short-term dietary consumption. Participants self-reported both intake frequency and portion size using standardized household measures provided within the EPIC-FFQ.

Dietary Pattern Analysis. Dietary patterns were identified using principal component analysis (PCA) with varimax rotation applied to standardized food-group intake variables, expressed as servings per day. Thirteen predefined food groups were included in the analysis. The adequacy of the data for PCA was assessed using the Kaiser–Meyer–Olkin (KMO) index and Bartlett’s test of sphericity. The number of retained dietary patterns was determined based on eigenvalues, inspection of the scree plot, and the interpretability of the extracted components. Three principal dietary patterns were retained, explaining a meaningful proportion of the total variance in dietary intake.

The Diet Quality Index during Pregnancy (DQI-P) was used to assess adherence to the recommended servings of grains, fruits, vegetables, folate, iron, and calcium, as well as to the recommended energy intake from fat [18,19]. Each component score of the DQI-P ranges from 0 to 10, except for fruits and vegetables, which are scored on a 0 to 20 scale. The sum of the DQI-P components yields a total score of 70, which is then transformed into a percentage.

### 2.3. Carotenoids Intake Assessment

To estimate individual carotenoids intake, dietary intake data obtained from the EPIC-FFQ were linked to the United States Department of Agriculture (USDA) FoodData Central database [20], which provides detailed information on the carotenoid composition of individual food items. Since the EPIC-FFQ does not directly provide values for specific carotenoids, a matching procedure between FFQ food items and USDA food composition entries was performed.

Initially, all unique food items from the EPIC-FFQ database were identified and matched to corresponding USDA food codes. A total of 201 USDA food entries were selected. The matching procedure was performed manually by a trained nutrition specialist to ensure the closest possible correspondence between Romanian dietary items included in the FFQ and available USDA food composition data. When appropriate, multiple USDA entries were assigned to a single FFQ food item in order to better reflect variability in food composition.

With this alignment made, we have merged the databases and filtered for the following compounds: Retinol, Lycopene, Carotene, β-Carotene, alpha-carotene, Vitamin E, Tocopherols and tocotrienols, Cryptoxanthin, Choline, total, Vitamin K (phylloquinone), Lutein + zeaxanthin, Vitamin D (D2 + D3), Vitamin A, RAE.

For FFQ items corresponding to multiple USDA food entries, intake values were calculated as the arithmetic mean of the matched components. For example, watermelon corresponds to two USDA entries with slightly different lycopene values (2709241 and 2709270); therefore, the final lycopene content assigned to watermelon represented the average value of the identified entries. Because the USDA database frequently reports lutein and zeaxanthin as combined values, these compounds were analyzed together throughout the study.

Because food composition values in the USDA database are reported per 100 g of food product, all values were standardized according to the portion sizes defined within the EPIC-FFQ database. Estimated intake values were subsequently calculated based on reported consumption frequency and portion size.

Given the absence of a comprehensive Romanian database that includes detailed carotenoid composition of foods, the USDA database was the most detailed publicly available resource for estimating individual carotenoids intake. Nevertheless, regional differences in food varieties, agricultural practices, and food processing methods may influence carotenoid composition and should therefore be considered when interpreting the findings.

The computational workflow, including Python 3.12 notebooks, merged databases, and intermediate processing steps, has been made publicly available through GitHub (see Data Availability Statement).

### 2.4. Statistical Analysis

Statistical analyses were performed using RStudio version 4.3.4 (R Foundation for Statistical Computing, Vienna, Austria). Descriptive statistics were used to summarize participant characteristics and dietary intake data. Continuous variables were presented as mean, standard deviation (SD), median, minimum, maximum, standard error (SE), and 95% confidence intervals (95% CI), whereas categorical variables were expressed as frequencies and percentages.

Differences in sociodemographic characteristics between groups were assessed using chi-square (χ^2^) tests for categorical variables. Differences in DQI-P scores according to educational level, income category, maternal age group, and parity were evaluated using one-way analysis of variance (ANOVA), followed by Tukey’s post-hoc tests when statistically significant overall differences were identified. Because several dietary intake variables showed non-normal distributions, associations between DQI-P scores and carotenoid intake values were assessed using Spearman correlation coefficients. Because total energy intake may influence estimated nutrient consumption, carotenoid intake values were energy-adjusted prior to regression analyses.

In addition, linear regression models were fitted using dietary patterns as the independent variables and carotenoids intakes as outcomes. Dietary patterns derived from principal component analysis were included as independent variables, whereas individual carotenoid intake values were treated as dependent variables. To reduce the risk of type I error due to multiple comparisons, *p*-values were adjusted using the Benjamini–Hochberg false discovery rate (FDR) procedure. Adjusted *p*-values < 0.05 were considered statistically significant.

### 2.5. Ethics

The current study was conducted in accordance with the Declaration of Helsinki and approved by the Ethics Committee of both Grigore T. Popa University of Medicine and Pharmacy (348/28 September 2023) and Elena Doamna Hospital of Obstetrics and Gynaecology, Iasi (1373/14 February 2023). Patient participation was voluntary and took place after receiving detailed information about the study’s purpose and completing the informed consent form. Informed consent was obtained from all subjects involved in the study.

## 3. Results

A total of 621 questionnaires were included in the final analysis. The study population consisted predominantly of women from rural areas (56.2%, *n* = 349), and slightly more than half of the participants were multiparous (51.2%, *n* = 318). Most participants were between 20 and 30 years of age (63.4%, *n* = 394). Regarding socioeconomic status, the largest proportion of participants reported low household income (33.5%, *n* = 208), followed by lower-middle income (30.8%, *n* = 191). Despite these findings, educational attainment was relatively high, with more than half of the participants holding either a bachelor’s degree (32.7%, *n* = 203) or a postgraduate degree (23.3%, *n* = 145).

Regarding prenatal care and nutrition, over two-thirds of the women did not receive any dietary counselling (67.8%, *n* = 421). Although multivitamin supplementation was common (67.1%, *n* = 417), supplementation with specific compounds was less frequent: only 44.3% (*n* = 275) of participants reported iron supplementation and 33.7% (*n* = 209) reported folic acid supplementation.

Assessment of diet quality using the Diet Quality Index for Pregnancy (DQI-P) indicated generally modest adherence to dietary recommendations. The largest proportion of participants obtained DQI-P scores below 40 points (36.6%, *n* = 227), followed by scores between 41–45 points (28.8%, *n* = 179) and 46–50 points (23.8%, *n* = 148). Only a relatively small proportion of participants achieved scores above 50 points. Detailed participant characteristics and DQI-P score distribution are presented in Table 1.

The dietary analysis revealed wide variations in the daily intake of specific fat-soluble vitamins, carotenoids, and choline among the participants. Regarding carotenoids, β-carotene exhibited the highest median intake at 2464 µg, with an Interquartile Range (IQR) of 2675 µg, followed by lycopene with a median of 1664 µg (IQR: 1622 µg). Lutein and zeaxanthin intakes had distributions of 908 µg (IQR: 560 µg), while α-carotene and β-cryptoxanthin intakes were lower, with medians of 615 µg and 121 µg, respectively. Table 2 presents the intake of carotenoids and related dietary compounds.

Total Vitamin A intake, measured as Retinol Activity Equivalents (RAE), was 853 µg (IQR: 697 µg), with pre-formed retinol accounting for 496 µg. For the remaining fat-soluble vitamins, participants reported median intakes of 5.58 µg (IQR: 2.19 µg) for Vitamin D, 11.7 mg (IQR: 7.68 mg) for Vitamin E, and 67 µg (IQR: 37.4 µg) for Vitamin K. Finally, the median dietary intake of choline was 393 mg (IQR: 149 mg). Maximum intake values varied substantially across participants, with β-carotene intake reaching 23,899 µg/day in some cases, suggesting marked heterogeneity in dietary patterns within the study population. Because intake distributions for carotenoids and related dietary compounds were positively skewed, results were summarized using medians and interquartile ranges. Detailed intake values are presented in Table 2.

Principal component analysis (PCA) was performed on a food-group intake dataset to identify predominant dietary patterns within the study population. Three factors were selected based on their eigenvalues, the scree plot, and interpretability, collectively accounting for a significant portion of the overall variance in dietary intake. The Kaiser–Meyer–Olkin (KMO) index was 0.61, indicating acceptable sampling adequacy for dimensionality reduction analysis, while Bartlett’s test of sphericity was statistically significant (*p* < 0.001), confirming sufficient correlations among variables for PCA.

Based on the magnitude and direction of factor loadings, three dietary patterns were identified. The first pattern, labelled “Energy-dense”, was characterized by high loadings for fats and oils, cereals and cereal products, and, to a lesser extent, sugars/preserves/snacks and non-alcoholic beverages. The second pattern, labelled “Mixed/animal”, showed high positive loadings for milk and milk products, fruit, meat and meat products, and nuts and seeds. The third pattern, labelled “Vegetable-meal”, was mainly characterised by vegetables and soups/sauces, suggesting a meal pattern centred on vegetable-based dishes. Detailed factor loadings for the identified dietary patterns are presented in Table 3.

**Figure 1:**
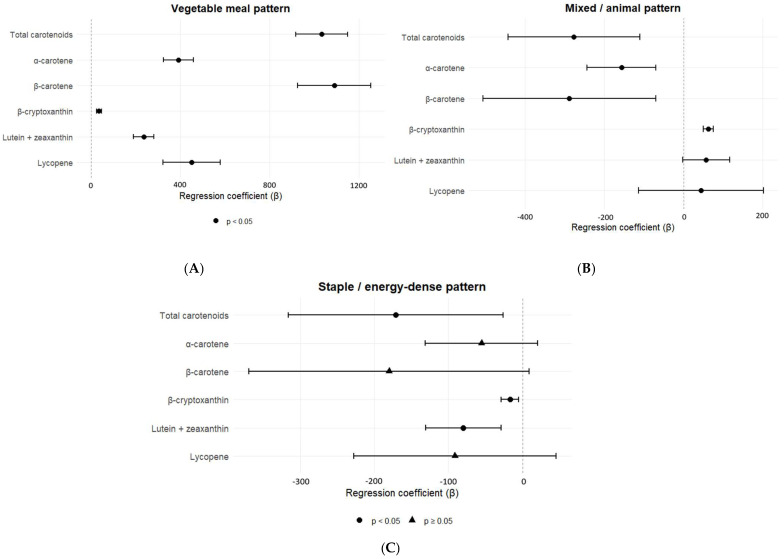
Forest plot of regression coefficients (β) and 95% confidence intervals for the associations between dietary patterns and energy-adjusted carotenoids intake. The dashed vertical line indicates the null value (β = 0). (**A**) vegetable-meal pattern; (**B**) mixed pattern, (**C**) energy-dense pattern.

Distinct and consistent associations were observed between the PCA-derived dietary patterns and intake of carotenoids and related dietary compounds (Figure 1).

The “Vegetable-meal” dietary pattern showed the strongest associations with carotenoids intake within the study population. Higher scores on this pattern were strongly associated with higher intakes of α-carotene, β-carotene, lutein and zeaxanthin, lycopene, and vitamin K. These associations remained statistically significant after adjustment for education level, household income, and area of residence, as well as correction for multiple testing using the false discovery rate (FDR) approach.

In contrast, compounds primarily derived from animal-based food sources demonstrated a different association profile. Retinol, vitamin A expressed as retinol activity equivalents (RAE), vitamin D, and choline were predominantly associated with the “Mixed/animal” dietary pattern. Higher adherence to this pattern was associated with significantly higher intake of these dietary compounds, reflecting their primary food sources and suggesting that mixed dietary patterns contribute substantially to variability in their intake.

The “Energy-dense” dietary pattern showed fewer significant associations overall. This dietary pattern was mainly associated with vitamin E and total tocopherol and tocotrienol intake, likely reflecting the contribution of fats and oils to these compounds. The associations between dietary patterns and carotenoids intake are shown in Table 4.

Regarding Diet Quality Index–Pregnancy (DQI-P), higher scores were significantly associated with higher intakes of most carotenoids and selected vitamins. In multivariable linear regression models adjusted for education, income, and area of residence, DQI-P scores showed strong positive associations with α-carotene, β-carotene, lutein/zeaxanthin, lycopene, retinol (vitamin A, RAE), vitamin D, vitamin K, and choline. These associations remained statistically significant after correction for multiple testing using the FDR method.

In contrast, no significant associations were observed between DQI-P scores and vitamin E or the sum of tocopherols and tocotrienols. This finding suggests that overall diet quality, as captured by the DQI-P, may more strongly reflect adherence to plant-rich dietary patterns and higher intake of carotenoids and related dietary compounds than intake of compounds predominantly derived from fats and oils. These results are presented in Table 5.

**Figure 2:**
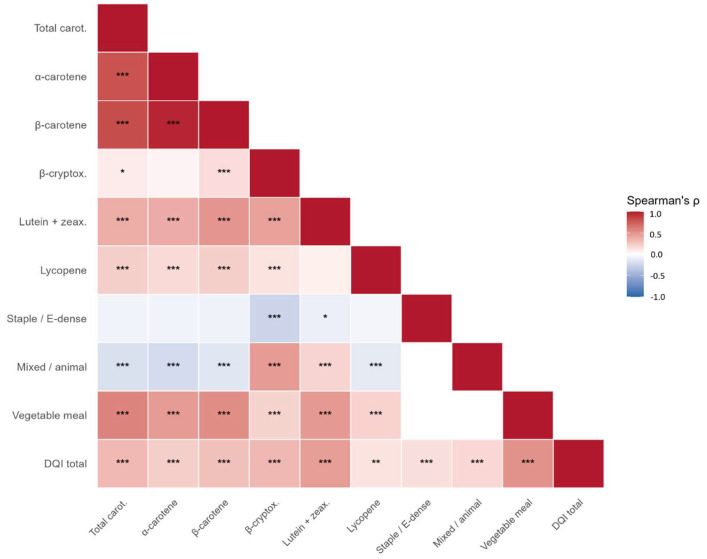
Heatmap of the relationship between DQI-P scores, dietary patterns and energy-adjusted carotenoids intake. Statistical significance: * *p* < 0.05; ** *p* < 0.01; *** *p* < 0.001.

Figure 2 illustrates the overall relationship between DQI-P scores, dietary patterns and carotenoids intake.

Additional details on nutrient intake and the complete correlation matrix are provided in the Supplementary Materials (Tables S1 and S2).

## 4. Discussion

The present study evaluated estimated dietary carotenoids intake among pregnant women from Romania and investigated its association with dietary patterns and overall diet quality. Carotenoids are among the most extensively studied plant-derived bioactive compounds due to their antioxidant and provitamin A activity [21]. In the present cohort, considerable interindividual variability was observed for all analyzed carotenoids and related dietary compounds, suggesting substantial heterogeneity in habitual dietary intake within the study population.

Among the evaluated carotenoids, β-carotene showed the highest median estimated intake at 2464 µg/day, with values ranging from 72 µg/day to 23,898 µg/day. β-carotene is one of the principal provitamin A carotenoids and contributes to vitamin A status through enzymatic conversion to retinol [22]. However, this conversion process is relatively inefficient in humans and may be influenced by multiple factors, including dietary composition, food matrix, overall vitamin A status, lipid intake, and genetic variability in BCMO1 activity [6,7]. Unlike excessive intake of preformed vitamin A, which has been associated with teratogenic effects and congenital malformations, provitamin A carotenoids such as β-carotene are generally considered safer because their conversion to retinol is physiologically regulated [7,23]. During pregnancy, maintaining an adequate vitamin A status is particularly important because fetal development depends on maternal retinol, retinyl esters, and provitamin A carotenoids [24].

Although no official recommended intake exists for individual carotenoids, previous studies have proposed a suggested intake range of 2–4 mg/day for β-carotene in adults, with potentially higher requirements during pregnancy and lactation [25]. In the present study, the median β-carotene intake was 2.46 mg/day, suggesting that only approximately half of the participants achieved this suggested intake range. Similar β-carotene intake values were previously reported in populations from Japan and the United Kingdom [26,27], whereas higher intake values were observed in studies conducted in the United States and Poland [28,29].

As a pigment, lycopene is bright red and is found at high concentrations in tomatoes and other red fruits and vegetables. Its high antioxidant capacity has made it a popular research molecule for various chronic diseases, including atherosclerosis, different types of cancer, and pregnancy-related conditions such as preeclampsia and intrauterine growth retardation [30,31,32,33]. Although no official recommended intake values have been established for lycopene, reported dietary intake varies considerably across populations. Previous studies reported average intakes ranging from approximately 0.5 to 5 mg/day in European populations and up to 10 mg/day in other populations, depending largely on consumption of tomatoes and tomato-based products [23]. Lycopene had a median intake of 1664 µg, with minimum and maximum intakes of 0 and 11,966 µg, respectively. Significantly higher intakes were observed by Litonjua et al. in the USA (7368.8 ± 3979.7 µg) and by Hamulka et al. in Poland (4.4 ± 3.3 mg/day) [28,29]. Differences in lycopene intake between Romanian pregnant women and other populations may partly reflect variations in dietary habits, food availability, socioeconomic conditions, and traditional dietary patterns. In particular, regional differences in tomato and tomato-based product consumption, seasonal food availability, and adherence to mixed dietary patterns may partly explain the lower intake observed in our population.

Lycopene intake is particularly important, given the high prevalence of maternal smoking during pregnancy, which we have also observed previously [34,35]. In line with these observations, the reported average lycopene intake varies significantly across regions, from approximately 1 mg/day in the United Kingdom to around 10 mg/day in the United States, highlighting regional differences in dietary habits and tomato product consumption [36].

Previous studies have proposed intake levels of approximately 10 mg/day for lutein and 2 mg/day for zeaxanthin [37]. Chemically, zeaxanthin is an isomer of lutein, differing only in the location of the double bond in the cyclic ring [37], which is why they are most often studied together. Dietary lutein and zeaxanthin had a median of 908 µg, lower than those reported by Litonjua et al. (2686.8 ± 1724.4 µg). A study of carotenoid distribution in infant brains found that lutein was the most common carotenoid in the occipital cortex, auditory cortex, hippocampus, and frontal lobe, areas linked to vision, hearing, memory, and executive functions, respectively [38]. Lutein and zeaxanthin are also the only 2 carotenoids that form the macular pigments in the eye by crossing the blood–retina barrier [39]. Moreover, lutein’s contribution to total carotenoid levels in infant brains is over half of the total carotenoid content, twice that in adults [39,40].

Dietary α-carotene and β-cryptoxanthin had lower intakes, with medians of 615 µg and 121 µg, respectively. Litonjua et al. reported similar findings, with a median of 878 ± 657.7 µg for alpha-carotene and 207.3 ± 130.9 µg for β-cryptoxanthin [28].

After principal component analysis, distinct associations between dietary patterns and estimated intake values were observed. The vegetable-based dietary pattern showed the strongest associations with intake of α-carotene, β-carotene, lutein and zeaxanthin, lycopene, and vitamin K. These findings are consistent with the role of vegetables as the primary dietary source of carotenoids. In contrast, the mixed/animal dietary pattern was predominantly associated with retinol, vitamin A expressed as retinol activity equivalents (RAE), vitamin D, and choline intake. The energy-dense dietary pattern showed fewer overall associations and was primarily linked to vitamin E and tocopherol intake.

Taken together, these findings suggest that overall dietary patterns are associated with variability in carotenoids intake and related dietary compounds during pregnancy. These findings should also be interpreted in the context of Romanian dietary practices, where regional food availability, socioeconomic disparities, and adherence to traditional dietary patterns may influence habitual carotenoids intake. While the DQI-P provides an overall measure of diet quality, PCA-based dietary patterns provide further insight into the combinations of food groups that drive variability in intake within the studied population. Additionally, the links between the vegetable-meal dietary pattern and carotenoids intake align with correlations between DQI-P scores and carotenoid levels, indicating that higher diet quality is associated with greater adherence to plant-focused dietary patterns.

Several limitations should be considered when interpreting these findings. First, dietary intake was assessed using a semiquantitative FFQ, which may be affected by recall bias, reporting inaccuracies, and limited ability to capture day-to-day dietary variability. Furthermore, because dietary intake was self-reported, socially desirable responses and inaccurate recall of habitual food consumption cannot be excluded. Although FFQs are widely used in dietary assessment research for estimating habitual dietary intake, they do not provide precise measurements of actual dietary consumption.

In addition, pregnancy is a dynamic period, and dietary intake may vary across gestational stages due to nausea, food aversions, cravings, supplement use, nutritional advice, or other pregnancy-related changes. Therefore, temporal changes in dietary habits throughout pregnancy may not have been fully captured. Although FFQs are widely used in dietary assessment research for estimating habitual dietary intake, they do not provide precise measurements of actual dietary consumption. Furthermore, FFQ-based methods are considered semiquantitative tools and may not accurately capture absolute dietary intake. Future studies should combine FFQ-derived estimates with repeated dietary recalls or biomarker-based validation methods.

Second, carotenoids intake was estimated using the USDA FoodData Central database because no comprehensive Romanian database that includes detailed carotenoid composition of foods is currently available. At present, no comprehensive Romanian food composition database including detailed carotenoid values is publicly available. Therefore, the USDA database represented the most comprehensive and standardized resource available for estimating individual carotenoids intake. Although the USDA database provides extensive carotenoid composition data, regional differences in food varieties, agricultural practices, seasonality, fortification, and food processing may influence the carotenoid composition of foods consumed in Romanian populations. Because carotenoid composition may vary with cultivar, agricultural practices, climate conditions, and food processing methods, the estimated intake values reported in the present study should be interpreted with caution. Consequently, the reported values should be interpreted as approximate estimates of dietary carotenoids intake rather than precise measurements of dietary consumption.

Additionally, the present study evaluated estimated dietary intake rather than circulating or tissue carotenoid concentrations. Future studies should investigate the relationship between FFQ-estimated carotenoids intake and biomarker-based validation methods. Residual confounding related to physical activity, smoking behavior, body composition, parity, and other lifestyle-related variables also cannot be excluded. Because the study used a cross-sectional design, causal relationships between dietary patterns and carotenoids intake cannot be inferred.

## 5. Conclusions

This study assessed the intake of individual carotenoids in pregnant women in Romania and examined how they relate to dietary patterns and overall diet quality. Using an adapted EPIC Food Frequency Questionnaire and a specialized estimation technique based on USDA food composition data, the research revealed significant variability among individuals, indicating diverse habitual dietary intakes during pregnancy. Among the carotenoids evaluated, β-carotene had the highest median intake, followed by lycopene, lutein and zeaxanthin, α-carotene, and β-cryptoxanthin, with notable differences in intake levels between participants.

The results revealed clear links between dietary patterns and nutritional profiles. The vegetable-based pattern had the most consistent association with increased intake of α-carotene, β-carotene, lutein, zeaxanthin, lycopene, and vitamin K, highlighting vegetables and plant foods as key sources of carotenoids. Conversely, the mixed/animal pattern was more strongly connected with retinol, vitamin A (measured as retinol activity equivalents, RAE), vitamin D, and choline intake. The energy-dense pattern showed fewer associations overall, mainly related to vitamin E and tocopherols. Additionally, higher DQI-P scores consistently correlated with increased consumption of carotenoids and related nutrients, suggesting that better adherence to dietary guidelines during pregnancy is linked to healthier nutritional profiles.

Overall, the current findings offer new insights into carotenoid consumption among pregnant women in Romania and enhance understanding of dietary variety and maternal nutritional habits in an Eastern European context. The study further demonstrates that estimating individual carotenoid compounds is feasible using an extant validated Food Frequency Questionnaire (FFQ) in conjunction with food composition data. However, these findings warrant cautious interpretation, as the EPIC-Norfolk FFQ was originally developed for a population in the United Kingdom, and dietary carotenoid estimates were derived from the USDA FoodData Central database rather than a Romanian-specific food composition dataset. Consequently, variations in food composition, cultivars, agricultural practices, and dietary behaviors may affect the precision and relevance of the intake estimations. Moreover, the semi-quantitative nature of FFQ-based dietary assessments and the cross-sectional study design are notable limitations that should be acknowledged. Future investigations should incorporate repeated dietary assessments, biomarker validation, and the development of population-specific food composition databases.

## Figures and Tables

**Table 1:** Sample characteristics.

Socioeconomic and Nutritional Characteristics		Counts	% of Total
Area of residence	Urban	272	43.80%
	Rural	349	56.20%
Parity	Multiparous	318	51.2%
	Nulliparous (first pregnancy)	303	48.8%
Age group	<20 years	52	8.40%
	20–30 years	394	63.40%
	>30 years	175	28.20%
Income	High	102	16.40%
	Low	208	33.50%
	Lower-middle	191	30.80%
	Upper-middle	120	19.30%
Formal education	Middle school	114	18.40%
	High school	159	25.60%
	Bachelor’s	203	32.70%
	Postgraduate	145	23.30%
Nutrition counseling	No	421	67.80%
	Yes	200	32.20%
Multivitamin supplements	No	204	32.90%
	Yes	417	67.10%
Iron supplements	No	346	55.70%
	Yes	275	44.30%
Folic acid supplements	No	412	66.30%
	Yes	209	33.70%
DQI-P groups	41–45	179	28.80%
	46–50	148	23.80%
	51–55	60	9.70%
	56–60	7	1.10%
	≤40	227	36.60%

**Table 2:** Estimated Intake of Carotenoids and Related Dietary Compounds.

Compound	Median	IQR	Min	Max
α-carotene (µg/day)	615	848	9.68	9914
β-carotene (µg/day)	2464	2675	72	23,898
β-cryptoxanthin (µg/day)	121	115	4.92	830
Lutein + zeaxanthin (µg/day)	908	560	131	11,836
Lycopene (µg/day)	1664	1622	7.32	11,965
Retinol (µg/day)	496	472	37.6	6946
Vitamin A, RAE (µg/day)	853	697	157	7552
Vitamin D (µg/day)	5.58	2.19	0.533	19.4
Vitamin E (mg/day)	11.7	7.68	3.44	78.4
Vitamin K (µg/day)	67	37.4	16.02	955
Choline (mg/day)	393	149	117.5	1095

Notes: Data are presented as median, interquartile range (IQR), minimum, and maximum values. Because distributions were markedly skewed, results were summarized using medians and interquartile ranges.

**Table 3:** Factor loading for dietary patterns.

Food Group	Dietary Pattern			Uniqueness
	Energy-Dense	Mixed	Vegetable-Meal	
Fats and oils	0.839			0.277
Cereals and cereal products	0.791			0.341
Sugars, preserves and snacks	0.369			0.831
Milk and milk products		0.655		0.561
Fruit		0.612	0.286	0.541
Non-alcoholic beverages	0.378	−0.584	0.356	0.389
Nuts and seeds	0.217	0.529		0.673
Meat and meat products	−0.213	0.463		0.737
Vegetables			0.728	0.468
Soups sauces			0.650	0.543
Potatoes			0.288	0.888
Eggs and egg dishes	0.253		0.269	0.826
Fish and fish products			0.215	0.952

**Table 4:** Association between dietary patterns and energy-adjusted carotenoids intake.

Outcome	Pattern	β (95% CI)	βstd	*p*
Lutein + zeaxanthin	Mixed/animal	57.21 (−1.73–116.14)	0.092	0.057
	Energy-dense	−79.79 (−130.60–−28.99)	−0.128	0.002
	Vegetable-meal	235.50 (189.12–281.88)	0.377	<0.001
Lycopene	Mixed/animal	43.91 (−113.70–201.52)	0.027	0.585
	Energy-dense	−91.21 (−227.57–45.15)	−0.055	0.189
	Vegetable-meal	450.82 (322.33–579.31)	0.272	<0.001
Total carotenoids	Mixed/animal	−276.12 (−442.52–−109.72)	−0.155	0.001
	Energy-dense	−171.03 (−315.77–−26.30)	−0.096	0.021
	Vegetable-meal	1033.79 (917.94–1149.63)	0.579	<0.001
α-carotene	Mixed/animal	−156.06 (−243.03–−69.09)	−0.166	<0.001
	Energy-dense	−55.38 (−131.34–20.58)	−0.059	0.153
	Vegetable-meal	390.69 (323.15–458.22)	0.415	<0.001
β-carotene	Mixed/animal	−287.56 (−505.17–−69.94)	−0.123	0.010
	Energy-dense	−180.11 (−369.10–8.87)	−0.077	0.062
	Vegetable-meal	1089.84 (926.20–1253.48)	0.468	<0.001
β-cryptoxanthin	Mixed/animal	62.19 (49.44–74.94)	0.416	<0.001
	Energy-dense	−16.95 (−28.72–−5.19)	−0.113	0.005
	Vegetable-meal	34.91 (23.68–46.14)	0.234	<0.001

Notes: β coefficients represent the change in energy-adjusted carotenoids intake associated with a one-unit increase in dietary pattern score. Models were adjusted for age, education, income, and area of residence. βstd = standardised beta coefficient.

**Table 5:** Association between diet quality (DQI) and energy-adjusted carotenoids intake.

DQI	β (95% CI)	βstd	*p*
Total carotenoids	97.26 (77.38–117.14)	0.365	<0.001
α-carotene	37.07 (26.30–47.84)	0.264	<0.001
β-carotene	107.27 (80.80–133.74)	0.308	<0.001
β-cryptoxanthin	7.40 (5.76–9.03)	0.331	<0.001
Lutein	30.10 (22.98–37.22)	0.322	<0.001
Lycopene	40.54 (20.77–60.32)	0.164	<0.001
Vitamin A, RAE	26.55 (18.78–34.32)	0.261	<0.001
Retinol	15.79 (8.23–23.35)	0.160	<0.001

Notes: β coefficients represent the change in energy-adjusted carotenoids intake per one-point increase in DQI score. Models adjusted for age, education, income, and area of residence. βstd = standardized beta.

## Data Availability

The coding lines and databases used in this study are openly available at https://github.com/Chiru-CatalinMihail/MITRAN_FOODDATA_2026, accessed on 16 June 2026.

## Supplementary Materials

The following supporting information can be downloaded at: https://www.mdpi.com/article/10.3390/nu18121999/s1, Table S1: Nutrient intake during pregnancy—general characterisation, Table S2: Correlation matrix.

## Abbreviations

FFQ	Food Frequency Questionnaire
EPIC-FFQ	European Prospective Investigation into Cancer and Nutrition Food Frequency Questionnaire
PCA	Principal Component Analysis
DQI-P	Diet Quality Index during Pregnancy
USDA	United States Department of Agriculture
RAE	Retinol Activity Equivalents
BCMO1	β-Carotene Monooxygenase 1
ROS	Reactive Oxygen Species
KMO	Kaiser–Meyer–Olkin
FDR	False Discovery Rate
IQR	Interquartile Range
SD	Standard Deviation
CI	Confidence Interval
ANOVA	Analysis of Variance
IU	International Units
FFQ-derived	Food Frequency Questionnaire-derived
βstd	Standardized Beta Coefficient
D2	Ergocalciferol
D3	Cholecalciferol
