# Supplementary material for: Evaluating Carotenoids Intake of Pregnant Women: A FFQ-Based Approach to Dietary Patterns
Source: Nutrients. 2026 Jun 19;18(12):1999. doi: 10.3390/nu18121999 (PMC13304667; doi:10.3390/nu18121999)
Supplement: Supplementary file 1 [file nutrients-18-01999-s001.zip › nutrients-4356361-supplementary.pdf]

Table S1. Nutrient intake during pregnancy – general characterization.

Nutrient	Mean	SE mean	95% CI mean lower bound	95% CI mean upper bound	Median	SD	IQR	Min	Max	25th percentile	50th percentile	75th percentile
Alcohol (g/day)	0.303	0.024	0.256	0.35	0	0.596	0.761	0	4.871	0	0	0.761
Total carbohydrate (g/day)	230.596	2.678	225.336	235.856	224.276	66.747	83.777	64.902	519.122	184.9	224.276	268.677
Cholesterol (mg/day)	379.992	5.764	368.673	391.312	369.676	143.642	166.293	59.419	1201.021	285.912	369.676	452.205
Fibre (NSP, g/day)	13.978	0.174	13.637	14.319	13.506	4.326	5.827	4.553	30.74	10.782	13.506	16.608
Fructose (g/day)	19.737	0.391	18.969	20.504	17.733	9.735	10.921	3.35	65.175	13.109	17.733	24.03
Galactose (g/day)	0.643	0.034	0.577	0.709	0.357	0.837	0.759	0	7.938	0	0.357	0.759
Glucose (g/day)	17.173	0.335	16.515	17.831	15.031	8.348	10.707	2.329	51.961	11.116	15.031	21.823
Energy (kcal/day)	1874.289	18.334	1838.284	1910.294	1832.442	456.889	567.632	910.871	3470.717	1555.98	1832.442	2123.612
Energy (kJ/day)	7880.775	77.056	7729.452	8032.098	7710.28	1920.232	2383.845	3812.108	14491.22	6546.2	7710.28	8930.045
Lactose (g/day)	15.008	0.264	14.49	15.526	15.875	6.576	3.502	0.548	47.959	14.185	15.875	17.687
Maltose (g/day)	2.295	0.071	2.155	2.436	1.851	1.781	2.669	0	16.421	0.962	1.851	3.631
Protein (g/day)	84.881	1.003	82.91	86.851	82.354	25.003	30.299	35.218	281.673	67.755	82.354	98.053
Starch (g/day)	130.108	1.761	126.65	133.566	128.065	43.884	56.794	26.586	404.151	98.624	128.065	155.419
Sucrose (g/day)	38.448	0.792	36.893	40.003	34.014	19.731	24.215	4.612	122.927	24.601	34.014	48.816
Nitrogen (g/day)	13.803	0.161	13.486	14.119	13.414	4.019	4.88	5.685	45.176	11.054	13.414	15.934
Total sµgars (g/day)	97.724	1.477	94.824	100.624	90.596	36.798	45.813	28.361	239.639	71.423	90.596	117.236
Total fat (g/day)	74.263	0.903	72.49	76.036	70.654	22.5	27.799	30.482	221.166	58.993	70.654	86.792
Monounsaturated fatty acids (g/day)	27.106	0.383	26.354	27.858	25.658	9.538	11.999	9.035	98.846	20.477	25.658	32.476
Polyunsaturated fatty acids (g/day)	12.55	0.196	12.166	12.934	11.746	4.873	5.467	3.96	65.263	9.339	11.746	14.806
Saturated fatty acids (g/day)	27.612	0.35	26.925	28.3	26.383	8.721	10.725	9.712	83.389	21.48	26.383	32.205
Calcium (mg/day)	834.32	9.711	815.25	853.39	824.064	241.991	262.814	222.237	1894.023	684.739	824.064	947.553
Chloride (mg/day)	4036.556	48.217	3941.868	4131.244	3934.412	1201.555	1573.621	1321.718	9610.906	3208.398	3934.412	4782.019
Copper (mg/day)	1.76	0.06	1.643	1.878	1.308	1.492	0.806	0.473	15.715	1.028	1.308	1.834
Iron (mg/day)	9.911	0.12	9.676	10.147	9.486	2.987	3.61	3.948	27.801	7.907	9.486	11.517
Folate (µg/day)	231.369	2.979	225.519	237.22	219.139	74.245	99.98	93.132	659.184	176.965	219.139	276.945
Iodine (µg/day)	131.158	1.609	127.999	134.317	127.427	40.091	44.896	44.45	333.232	104.683	127.427	149.579
Potassium (mg/day)	2805.34	27.991	2750.372	2860.309	2749.801	697.533	870.366	1173.864	5797.866	2320.273	2749.801	3190.638
Magnesium (mg/day)	246.317	2.503	241.401	251.233	238.736	62.384	80.934	107.678	578.365	201.392	238.736	282.325

Manganese (mg/day)	2.363	0.039	2.287	2.438	2.121	0.961	0.984	0.547	7.117	1.74	2.121	2.724
Sodium (mg/day)	2676.562	32.815	2612.12	2741.004	2597.477	817.741	1057.855	898.58	6264.103	2116.635	2597.477	3174.49
Niacin (mg/day)	21.604	0.315	20.987	22.222	20.536	7.839	8.839	5.925	87.77	16.492	20.536	25.33
Phosphorus (mg/day)	1284.067	13.118	1258.305	1309.828	1252.672	326.901	429.275	513.471	3027.827	1047.682	1252.672	1476.957
Riboflavin (mg/day)	1.903	0.037	1.83	1.975	1.676	0.919	1.068	0.487	8.893	1.3	1.676	2.368
Selenium (µg/day)	81.751	1.028	79.732	83.769	80.855	25.614	31.12	22.85	224.299	64.164	80.855	95.284
Thiamin (mg/day)	1.325	0.015	1.295	1.355	1.286	0.377	0.487	0.535	3.201	1.062	1.286	1.549
Vitamin B12 (µg/day)	8.805	0.395	8.03	9.58	6.079	9.832	6.065	0.439	101.534	3.484	6.079	9.548
Vitamin B6 (mg/day)	1.888	0.021	1.847	1.929	1.821	0.522	0.744	0.768	4.495	1.503	1.821	2.247
Vitamin C (mg/day)	93.621	2.282	89.139	98.102	80.2	56.872	51.749	7.651	420.479	57.849	80.2	109.598
Zinc (mg/day)	8.898	0.107	8.688	9.108	8.597	2.664	3.645	3.59	24.615	6.906	8.597	10.551
Retinol (µg/day)	711.206	27.475	657.25	765.162	495.912	684.683	467.699	37.617	6946.023	323.071	495.912	790.77
Vitamin A, RAE (µg/day)	1026.8	28.849	970.146	1083.454	853.367	718.92	691.14	157.035	7552.592	566.13	853.367	1257.27
Vitamin D (µg/day)	5.82	0.097	5.628	6.011	5.579	2.429	2.162	0.533	19.392	4.467	5.579	6.629
Vitamin E (mg/day)	13.394	0.306	12.793	13.995	11.65	7.625	7.515	3.443	78.382	8.522	11.65	16.037
Vitamin K (µg/day)	73.744	1.941	69.932	77.556	67.009	48.375	37.358	16.029	954.74	51.257	67.009	88.616
Choline (mg/day)	402.16	4.918	392.502	411.818	392.976	122.562	148.437	117.58	1095.476	314.836	392.976	463.273
Alpha-carotene (µg/day)	1055.795	38.256	980.667	1130.923	615.102	953.342	846.103	9.688	9913.746	400.576	615.102	1246.679
Beta-carotene (µg/day)	3172.877	95.385	2985.56	3360.194	2464.63	2376.983	2648.626	72.156	23898.66	1438.799	2464.63	4087.425
Beta-cryptoxanthin (µg/day)	163.196	6.055	151.304	175.087	120.825	150.898	114.435	4.929	829.786	74.933	120.825	189.368
Lutein + zeaxanthin (µg/day)	1001.816	25.529	951.682	1051.951	907.89	636.19	554.996	130.943	11836.48	655.332	907.89	1210.328
Lycopene (µg/day)	1937.316	67.889	1803.996	2070.636	1664.807	1691.785	1617.379	7.321	11965.64	760.968	1664.807	2378.347

Table S2. Correlation matrix.

	total_carot_adj	alpha_carotene_adj	beta_carotene_adj	lutein_adj	lycopene_adj	cryptox_adj	lutein_zeax_adj	Staple energy dense component	Mixed_fresh_animal_component	Vegetable_meal_component	DQI_P_total
total_carot_adj	1 (NA)	0.781 (<0.001)	0.823 (<0.001)	0.456 (<0.001)	0.255 (<0.001)	0.163 (<0.001)	0.456 (<0.001)	0.183 (<0.001)	-0.061 (0.128)	0.64 (<0.001)	0.432 (<0.001)
alpha_carotene_adj	0.781 (<0.001)	1 (NA)	0.961 (<0.001)	0.464 (<0.001)	0.197 (<0.001)	0.123 (0.002)	0.464 (<0.001)	0.15 (<0.001)	-0.076 (0.058)	0.56 (<0.001)	0.346 (<0.001)
beta_carotene_adj	0.823 (<0.001)	0.961 (<0.001)	1 (NA)	0.563 (<0.001)	0.258 (<0.001)	0.238 (<0.001)	0.563 (<0.001)	0.159 (<0.001)	-0.014 (0.736)	0.6 (<0.001)	0.4 (<0.001)
lutein_adj	0.456 (<0.001)	0.464 (<0.001)	0.563 (<0.001)	1 (NA)	0.147 (<0.001)	0.5 (<0.001)	1 (<0.001)	0.133 (<0.001)	0.289 (<0.001)	0.532 (<0.001)	0.531 (<0.001)
lycopene_adj	0.255 (<0.001)	0.197 (<0.001)	0.258 (<0.001)	0.147 (<0.001)	1 (NA)	0.159 (<0.001)	0.147 (<0.001)	0.181 (<0.001)	-0.019 (0.645)	0.294 (<0.001)	0.224 (<0.001)
cryptox_adj	0.163 (<0.001)	0.123 (0.002)	0.238 (<0.001)	0.5 (<0.001)	0.159 (<0.001)	1 (NA)	0.5 (<0.001)	-0.065 (0.108)	0.551 (<0.001)	0.28 (<0.001)	0.415 (<0.001)
lutein_zeax_adj	0.456 (<0.001)	0.464 (<0.001)	0.563 (<0.001)	1 (<0.001)	0.147 (<0.001)	0.5 (<0.001)	1 (NA)	0.133 (<0.001)	0.289 (<0.001)	0.532 (<0.001)	0.531 (<0.001)
Staple_energy_dense_component	0.183 (<0.001)	0.15 (<0.001)	0.159 (<0.001)	0.133 (<0.001)	0.181 (<0.001)	-0.065 (0.108)	0.133 (<0.001)	1 (NA)	-0.006 (0.876)	-0.003 (0.939)	0.146 (<0.001)
Mixed_fresh_animal_component	-0.061 (0.128)	-0.076 (0.058)	-0.014 (0.736)	0.289 (<0.001)	-0.019 (0.645)	0.551 (<0.001)	0.289 (<0.001)	-0.006 (0.876)	1 (NA)	0.002 (0.950)	0.184 (<0.001)
Vegetable_meal_component	0.64 (<0.001)	0.56 (<0.001)	0.6 (<0.001)	0.532 (<0.001)	0.294 (<0.001)	0.28 (<0.001)	0.532 (<0.001)	-0.003 (0.939)	0.002 (0.950)	1 (NA)	0.513 (<0.001)
DQI_P_total	0.432 (<0.001)	0.346 (<0.001)	0.4 (<0.001)	0.531 (<0.001)	0.224 (<0.001)	0.415 (<0.001)	0.531 (<0.001)	0.146 (<0.001)	0.184 (<0.001)	0.513 (<0.001)	1 (NA)
total_carot_adj	1 (NA)	0.792 (<0.001)	0.82 (<0.001)	0.378 (<0.001)	0.222 (<0.001)	0.091 (0.024)	0.378 (<0.001)	-0.07 (0.083)	-0.191 (<0.001)	0.575 (<0.001)	0.328 (<0.001)
alpha_carotene_adj	0.792 (<0.001)	1 (NA)	0.966 (<0.001)	0.39 (<0.001)	0.173 (<0.001)	0.05 (0.210)	0.39 (<0.001)	-0.075 (0.062)	-0.219 (<0.001)	0.47 (<0.001)	0.232 (<0.001)
beta_carotene_adj	0.82 (<0.001)	0.966 (<0.001)	1 (NA)	0.494 (<0.001)	0.226 (<0.001)	0.16 (<0.001)	0.494 (<0.001)	-0.076 (0.059)	-0.15 (<0.001)	0.526 (<0.001)	0.293 (<0.001)
lutein_adj	0.378 (<0.001)	0.39 (<0.001)	0.494 (<0.001)	1 (NA)	0.069 (0.085)	0.443 (<0.001)	1 (<0.001)	-0.102 (0.011)	0.198 (<0.001)	0.476 (<0.001)	0.459 (<0.001)

lycopene_adj	0.222 (<0.001)	0.173 (<0.001)	0.226 (<0.001)	0.069 (0.085)	1 (NA)	0.134 (<0.001)	0.069 (0.085)	-0.061 (0.131)	-0.133 (<0.001)	0.207 (<0.001)	0.129 (0.001)
cryptox_adj	0.091 (0.024)	0.05 (0.210)	0.16 (<0.001)	0.443 (<0.001)	0.134 (<0.001)	1 (NA)	0.443 (<0.001)	-0.263 (<0.001)	0.468 (<0.001)	0.204 (<0.001)	0.331 (<0.001)
lutein_zeax_adj	0.378 (<0.001)	0.39 (<0.001)	0.494 (<0.001)	1 (<0.001)	0.069 (0.085)	0.443 (<0.001)	1 (NA)	-0.102 (0.011)	0.198 (<0.001)	0.476 (<0.001)	0.459 (<0.001)
Staple_energy_dense_component	-0.07 (0.083)	-0.075 (0.062)	-0.076 (0.059)	-0.102 (0.011)	-0.061 (0.131)	-0.263 (<0.001)	-0.102 (0.011)	1 (NA)	-0.006 (0.876)	-0.003 (0.939)	0.146 (<0.001)
Mixed_fresh_animal_component	-0.191 (<0.001)	-0.219 (<0.001)	-0.15 (<0.001)	0.198 (<0.001)	-0.133 (<0.001)	0.468 (<0.001)	0.198 (<0.001)	-0.006 (0.876)	1 (NA)	0.002 (0.950)	0.184 (<0.001)
Vegetable_meal_component	0.575 (<0.001)	0.47 (<0.001)	0.526 (<0.001)	0.476 (<0.001)	0.207 (<0.001)	0.204 (<0.001)	0.476 (<0.001)	-0.003 (0.939)	0.002 (0.950)	1 (NA)	0.513 (<0.001)
DQI_P_total	0.328 (<0.001)	0.232 (<0.001)	0.293 (<0.001)	0.459 (<0.001)	0.129 (0.001)	0.331 (<0.001)	0.459 (<0.001)	0.146 (<0.001)	0.184 (<0.001)	0.513 (<0.001)	1 (NA)
